# Changes in the prevalence of hepatitis B virus and its related factors in Inner Mongolia between 2006 and 2020

**DOI:** 10.3389/fpubh.2025.1533938

**Published:** 2025-04-08

**Authors:** Xuejie Ding, Zhongbing Zhang, Cheng Li, Hui Song, Shuna Ding, Yu Zhou, Xianyun Ren, Fei Hou, Xia Wen, Chunyan Li, Libo Wang, Junqing Ma, Liwei Zhang, Yan Wang, Shuling Wang, Chunmei Geng, Shan Wu, Junmei Gu, Xiaoling Tian, Qingbin Lu

**Affiliations:** ^1^School of Public Health, Inner Mongolia Medical University, Hohhot, China; ^2^Inner Mongolia Center for Disease Control and Prevention, Hohhot, China; ^3^North China University of Science and Technology Affiliated Hospital, Tangshan, China; ^4^School of Public Health, BaoTou Medical College, Baotou, China; ^5^Center for Disease Control and Prevention of Hohhot, Hohhot, China; ^6^Center for Disease Control and Prevention of Baotou, Baotou, China; ^7^Center for Disease Control and Prevention of Hulunbeier, Hulunbeier, China; ^8^Center for Disease Control and Prevention of Hinggan League, Hinggan League, China; ^9^Center for Disease Control and Prevention of Tongliao, Tongliao, China; ^10^Center for Disease Control and Prevention of Chifeng, Chifeng, China; ^11^Center for Disease Control and Prevention of Xilingol League, Xilingol League, China; ^12^Center for Disease Control and Prevention of Erdos, Erdos, China; ^13^Center for Disease Control and Prevention of Wuhai, Wuhai, China; ^14^Center for Disease Control and Prevention of Bayannur, Bayannur, China; ^15^Center for Disease Control and Prevention of Alxa, Alxa, China; ^16^Center for Disease Control and Prevention of Ulanqab, Ulanqab, China; ^17^Laboratory Science and Technology, School of Public Health, Peking University, Beijing, China

**Keywords:** infectious disease, hepatitis B vaccine, cross-sectional study, HBV prevalence, China

## Abstract

**Objective:**

This study aimed to compare the prevalence of serum hepatitis B virus (HBV) markers in Inner Mongolia between 2006 and 2020.

**Methods:**

The same sampling process was used in investigations conducted in 2006 and 2020. A multi-stage stratified random sampling method was used to select subjects aged 1–60 years old from 12 cities in Inner Mongolia. Blood samples were collected to detect serological HBV markers including hepatitis B surface antigen (HBsAg), hepatitis B surface antibody (HBsAb), and hepatitis B core antibody (HBcAb). The prevalence of serum biomarkers of hepatitis B, standardized by age and sex, was analyzed and compared between 2006 and 2020.

**Results:**

There were 6,304 subjects in 2006 and 6,500 in 2020. The prevalence of HBsAg was higher in 2006 than in 2020 (standardized 4.11% vs. 2.75%, *p* < 0.001). The results were observed for the serum biomarkers of HBsAb (standardized 41.40% vs. 42.14%, *p* = 0.39) between 2006 and 2020, as well as HBcAb (standardized 22.91% vs. 20.15%, *p* < 0.001). The hepatitis B vaccine (Hep B vaccine) provides protection against HBV infection. In 2006, the proportions of timely birth dose (TBD) and 3-dose Hep B vaccine coverage for individuals aged 1–14 years were 80.57 and 89.35%, respectively. By 2020, these proportions increased to 97.43 and 96.97%, respectively.

**Conclusion:**

The prevalence of HBsAg decreased significantly from 2006 to 2020 in Inner Mongolia, suggesting that the Hep B vaccine has made remarkable progress in safeguarding the population against hepatitis B infection.

## 1 Introduction

Hepatitis B virus (HBV) infection remains a pressing public health priority. HBV can cause acute infection and progress to chronic infection in the absence of standardized antiviral treatment, even leading to liver cirrhosis (1). Several studies have demonstrated that the prevalence of HBV infection varies significantly by geographical region, with higher incidence rates observed in Africa and various parts of the Asia-Pacific region (2). It is generally recognized that the differences in prevalence among countries are primarily attributed to disparities in routine hepatitis B vaccine (Hep B vaccine) policies among nations. These differences subsequently affect immunity. Despite the vaccination recommendations of the World Health Organization (WHO) and national immunization guidelines, gaps in Hep B vaccine coverage remain among healthcare workers (3). In 2015, the WHO launched a strategy to eliminate hepatitis as a public health threat by 2030. However, if the current situation remains unchanged, annual global deaths from HBV are projected to increase by 39% between 2015 and 2030 (4). In China, which has the highest burden of HBV infection, HBV-related liver diseases account for 30% of the worldwide mortality caused by HBV infection (5, 6).

Inner Mongolia, an autonomous region in northern China, has a total area of 1.183 million square kilometers and a population of 25.396 million. It stretches over a long distance from east to west and shares borders with numerous provinces and countries (7). The Hep B vaccine has been used in Inner Mongolia for many years. The prevalence of HBsAg in subjects aged 1–16 years old in Inner Mongolia has dropped to a relatively low level, while that in subjects over 20 years of age remains at a relatively high level (8). However, few studies have evaluated the effects of this vaccine in Inner Mongolia after its use. This study analyzed the prevalence of three HBV serologic results (HBsAg, HBsAb, and HBcAb) in 2006 compared to 2020. This study aimed to demonstrate the changes in local risk factors for HBV infection after vaccination and to provide intervention measures to achieve the goal of eliminating HBV by 2030.

## 2 Methods

### 2.1 Study site

Inner Mongolia is located in North China with a gross domestic product (GDP) of 2,315.9 billion yuan and a per capita GDP of 96,474 yuan. It has 12 prefecture-level administrative regions (Hohhot, Chifeng, Ordos, Ulanqab, Baotou, Bayannur, Hulunbuir, Wuhai, Tongliao, Xilin Gol, Hinggan League, and Alxa) and 103 county-level administrative units, with a permanent population of 24 million.

### 2.2 Subjects

The same sampling process was used in the investigations conducted in 2006 and 2020, in accordance with the requirements of the national viral hepatitis immune evaluation survey. A multi-stage stratified random sampling method was utilized to select subjects aged 1–60 years old from 12 cities in Inner Mongolia. First, one county was randomly selected from each of the 12 cities. Second, two committees were randomly chosen from each of the 12 counties based on the Statistical Yearbook of Inner Mongolia. Each resident was assigned a unique code based on their region and divided into four age groups, including 1–4 years, 5–14 years, 15–29 years, and 30–60 years. Third, simple random sampling was performed within each age group using the Statistical Yearbook of Inner Mongolia. Considering the 10% rejection rate for the survey, the sample size was estimated using a multistage sampling calculation method. In 2006, the total sample size was 6,304, distributed across four age groups as follows: 795, 2,041, 1,266, and 2,202, respectively. In 2020, the sample size was 6,500, with the four age groups comprising 926, 1,297, 1,367, and 2,910, respectively. The sample size was estimated using the following formula.

$$n=\;\frac{z^{2}\alpha /2\times p\times \left(1-p\right)}{\delta^{2}}\times \mathrm{deff}$$

### 2.3 Information collecting

Professionally trained personnel collected and completed the questionnaire. The contents of the questionnaire included general demographic characteristics (gender, age, and occupation), hepatitis history, and hepatitis B vaccination history based on the vaccination certificate.

### 2.4 Sample collection and laboratory detection

Venous blood was collected, and serum was separated naturally, then frozen at −20°C for examination. In 2006, an enzyme-linked immunoassay was used as the detection method. The diagnostic kits used in this study were provided by Xiamen InTec PRODUCTS, Inc. In 2020, a chemiluminescent immunoassay was used as the detection method. The diagnostic kits used in this study were provided by Abbott Laboratories (United States). All tests were performed according to the kit instructions. HBV infection was defined as positive for either HBsAg or HBcAb (9, 10). The experimental samples were collected in 2006 and 2020. The laboratory testing times of the samples were from 2006 to 2008 and from 2020 to 2022.

### 2.5 Statistical analysis

We calculated the prevalence of HBV markers and 95% confidence intervals (CIs) based on a binomial distribution. We also calculated the proportion of timely birth dose (TBD) and 3-dose Hep B vaccine in children aged 1 to 14 years. The prevalence of HBV markers and Hep B vaccine proportions between 2006 and 2020 was compared using the chi-square test. The same statistical method was used to compare the prevalence of HBV markers and Hep B vaccine proportions among different subjects in the same year survey. A multivariable logistic regression model was used to determine the factors related to the prevalence of HBV markers and to calculate the odds ratio (OR). Factors with an OR > 1 were considered risk factors. Excel (2016) was used to collate the survey data, and STATA software (version 17) was used for analysis. The significance threshold was set at a *p*-value of <0.05.

### 2.6 Ethical considerations

This study was approved by the Ethics Committee of the Inner Mongolia CDC and conducted in accordance with the national ethical code. This survey was conducted in accordance with the ethical guidelines of the Declaration of Helsinki. Participants were informed of the purpose of the survey and informed consent was obtained before the samples were collected. The data will be kept confidential.

## 3 Results

### 3.1 Basic information

A total of 6,304 subjects aged 1–60 years were investigated in 2006. Mongol nationality accounted for 1,539 (24.41%), as shown in Table 1. A total of 6,500 subjects aged 1–60 years were included in 2020, of which 2,972 (45.72%) were male. The Mongolian ethnicity accounted for 1,863 (28.66%), and the Han ethnicity accounted for 4,453 (68.51%); urban, rural, and pastoral areas accounted for 1,802 (27.72%), 2,069 (31.83%), and 2,629 (40.45%), respectively. The subjects aged 30–60 years made up the largest proportion and accounted for 2,910 (44.77%).

**Table 1:** Comparison of the composition of the subjects between 2006 and 2020.

Characteristics	2006		2020		
	No.	Proportion (%)	No.	Proportion (%)	*P*
Sex					0.37
Male	2,932	46.51	2,972	45.72	
Female	3,372	53.49	3,528	54.28	
Ethnicity					<0.001
Han	4,173	66.20	4,453	68.51	
Mongol	1,539	24.41	1863	28.66	
Other	592	9.39	184	2.83	
Age, years					<0.001
1–4	795	12.61	926	14.25	
5–14	2041	32.38	1,297	19.95	
15–29	1,266	20.08	1,367	21.03	
30–60	2,202	34.93	2,910	44.77	
Region					<0.001
Urban	2,352	37.31	1802	27.72	
Rural	1942	30.81	2069	31.83	
Pastoral	2010	31.88	2,629	40.45	
Education level					<0.001
Primary	511	14.73	655	15.31	
Junior	1,441	41.55	1,175	27.47	
Senior	874	25.21	974	22.77	
College	642	18.51	1,473	34.44	
Occupation					<0.001
Student	949	27.36	347	28.63	
Peasant-worker	741	21.37	1,212	28.34	
Teacher/Office	441	12.72	765	17.89	
Healthcare workers	144	4.15	257	6.01	
Other	1,193	34.40	1,696	39.65	

The proportion of TBD of the Hep B vaccine was 97.43% (95%CI: 96.73–98.08%), and 3-dose vaccination was 96.97% (95%CI: 96.20–97.67%) in 2020, which was higher than that in 2006 (*p* < 0.001). In both the TBD and 3-dose vaccination, the proportion was higher in urban areas than in rural or pastoral areas, as shown in Tables 2, 3 (*p* < 0.001). The proportion of patients with TBD was 99.68% (95%CI: 98.99–99.90%), and the 3-dose was 99.46% (95%CI: 98.71–99.78%) under 5 years of age, higher than those in other ages (*p* < 0.001).

**Table 2:** Comparison of the proportion of TBD of Hep B vaccine among different subjects aged 1 to 14 years between 2006 and 2020.

Characteristics		2006			2020		*P*
	No.	Proportion (%)	95% CI	No.	Proportion (%)	95% CI	
Sex							<0.001
Male	1,144	79.50	77.33–81.51	1,106	97.53	96.45–98.29	
Female	1,141	81.67	79.56–83.62	1,015	97.31	96.14–98.14	
Age, years							<0.001
≤4	701	88.18	85.74–90.25	923	99.68	98.99–99.90	
5–10	1,081	84.45	82.36–86.34	806	98.77	97.74–99.35	
11–14	503	66.10	62.65–69.38	392	90.11	86.92–92.59	
Ethnicity							<0.001
Han	1,492	80.13	78.25–81.88	1,422	97.20	96.21–97.93	
Mongol	536	76.24	72.95–79.25	647	98.03	96.63–98.85	
Other	257	94.83	91.45–96.92	52	96.30	85.97–99.10	
Region							<0.001
Urban	1,014	94.94	93.63–96.26	683	99.71	99.30–100.1	
Rural	669	72.25	69.36–75.14	725	94.52	92.91–96.13	
Pastoral	602	71.50	68.44–74.55	713	98.34	97.41–99.28	

**Table 3:** Comparison of the proportion of 3-dose Hep B vaccine among different subjects aged 1 to 14 years between 2006 and 2020.

Characteristics		2006			2020		*P*
	No.	Proportion (%)	95% CI	No.	Proportion (%)	95% CI	
Sex							<0.001
Male	1,273	88.46	86.71–90.02	1,100	97.00	95.83–97.85	
Female	1,261	90.26	88.59–91.71	1,011	96.93	95.69–97.82	
Age, years							<0.001
≤4	770	96.86	95.38–97.87	921	99.46	98.71–99.78	
5–10	1,202	93.91	92.45–95.09	799	97.92	96.67–98.70	
11–14	562	73.85	70.60–76.85	391	89.89	86.67–92.39	
Ethnicity							<0.001
Han	1,648	88.51	86.98–89.88	1,413	96.58	95.52–97.40	
Mongol	621	88.34	85.74–90.51	646	97.88	96.45–98.74	
Other	265	97.79	95.14–99.01	52	96.29	85.97–99.10	
Region							<0.001
Urban	1,025	95.97	94.79–97.15	676	98.69	97.83–99.54	
Rural	800	86.39	84.18–88.61	722	94.13	92.91–96.14	
Pastoral	709	84.20	65.01–70.68	712	98.21	97.24–99.18	

### 3.2 Prevalence of HBsAg

**Figure 1:**
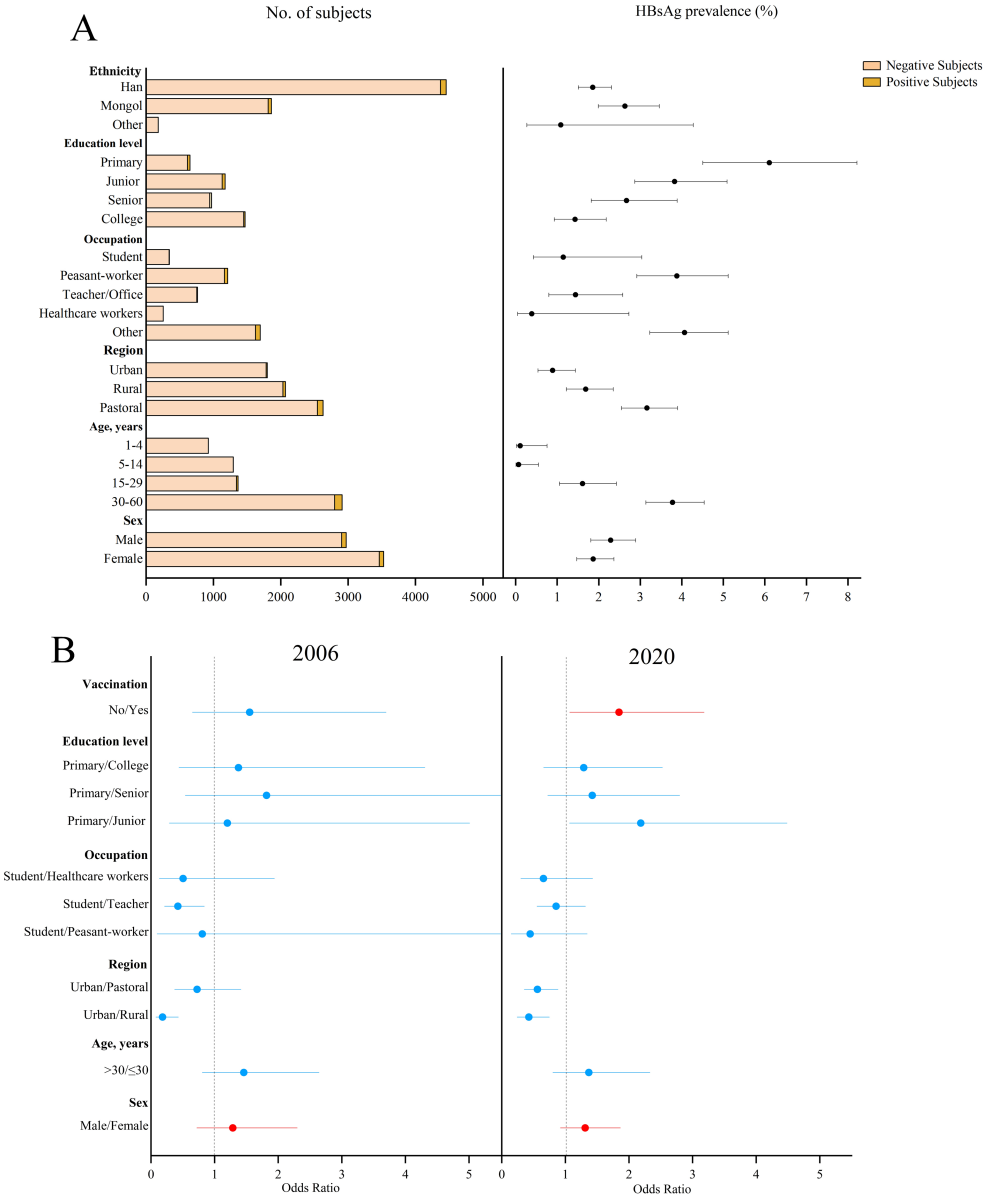
Prevalence of HBsAg in different subjects in 2020 and the possible factors related to the prevalence of HBsAg in 2006 and 2020 in Inner Mongolia. **(A)** The positive result and 95% CI for the prevalence of HBsAg in different subjects of Inner Mongolia in 2020. **(B)** The possible factors related to HBsAg prevalence in 2006 and 2020, including red means risk factors and blue means protective factors or vitiating factors.

The prevalence of HBsAg showed a downward trend over the period (*p* = 0.012). The prevalence of HBsAg was 2.74% (95%CI: 2.34–3.15%) in 2006 and 2.06% (95%CI: 1.72–2.41%) in 2020 (*p* = 0.012; standardized 4.11% vs. 2.75%) in Figure 1. The 2020 survey results showed that the prevalence of HBsAg was 2.29% (95%CI: 1.81–2.89%) in male participants and 1.87% (95%CI: 1.47–2.37%) in female participants (*p* = 0.24; standardized 1.18% vs. 0.90%, *p* = 0.28). The Mongolian ethnicity had a higher HBsAg prevalence of 2.63% (95%CI: 1.99–3.46%) than the Han ethnicity (1.86, 95%CI: 1.51–2.31%, *p* = 0.10). The prevalence of HBsAg in urban areas was 0.89% (95%CI: 0.54–1.44%), which was significantly lower than that in rural and pastoral areas (1.69 and 3.16%, respectively, both *p* < 0.001). Among the different age groups, the lowest prevalence of HBsAg was 0.07% (95%CI: 0.01–0.55%) in children aged 5–14 years. Among the subjects aged 15–60 years, the prevalence of HBsAg was inversely proportional to the level of education. The highest prevalence of HBsAg was 6.11% (95%CI: 4.51–8.22%) in subjects with primary school education or below, and 3.88% (95%CI: 2.92–5.12%) in farmers. The possible factors related to the prevalence of HBsAg were male sex (OR = 1.29, 95%CI: 0.72–2.30) in 2006 and male sex (OR = 1.31, 95%CI: 0.93–1.86) and hepatitis B vaccination history (OR = 1.84, 95%CI: 1.07–3.18) in 2020.

### 3.3 Prevalence of HBsAb

**Figure 2:**
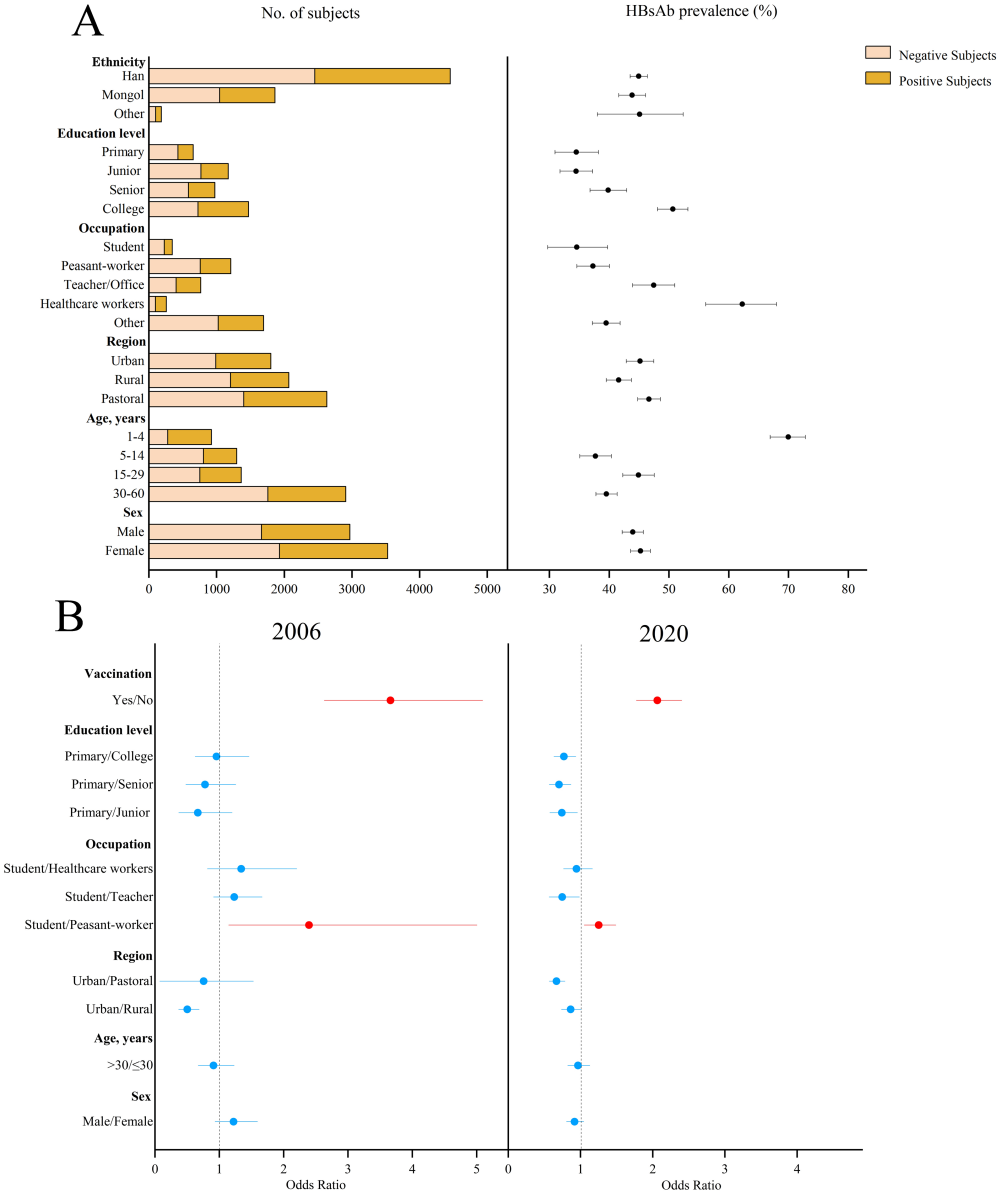
Prevalence of HBsAb in different subjects in 2020 and the possible factors related to HBsAb prevalence in 2006 and 2020 in Inner Mongolia. **(A)** The positive result and 95% CI for the prevalence of HBsAb in different subjects of Inner Mongolia in 2020. **(B)** The possible factors related to HBsAb prevalence in 2006 and 2020, including red means risk factors and blue means protective factors or vitiating factors.

The prevalence of HBsAb showed an upward trend over the period (*p* < 0.001). The prevalence of HBsAb in the subjects aged 1–60 years was 44.76% (95%CI: 43.52–45.98%) in 2006 and 44.65% (95%CI: 43.44–45.86%) in 2020 (*p* < 0.001) in Figure 2. There were 41.40 and 42.14% (*p* = 0.39), respectively, after standardization by age and sex using the entire Inner Mongolian population. The highest prevalence of HBsAb was 69.98% (95%CI: 66.94–72.85%) in children under 5 years of age, while the lowest prevalence of HBsAb was 37.70% (95%CI: 35.10–40.38%) in those aged 5–14 years. Rural areas had the lowest prevalence of HBsAb (41.61, 95%CI: 39.51–43.75%, *p* = 0.002) compared to the other two regions. The prevalence of HBsAb was the highest among healthcare workers (62.26, 95%CI: 56.15–68.00%) and the lowest among students (34.58, 95%CI: 29.75–39.76%). Occupation (student vs. peasant-worker, OR = 2.39, 95%CI: 1.15–5.00) and hepatitis B vaccination history (OR = 3.66, 95%CI: 2.63–5.09) were related to the prevalence of HBsAb in 2006 and 2020.

### 3.4 Prevalence of HBcAb

**Figure 3:**
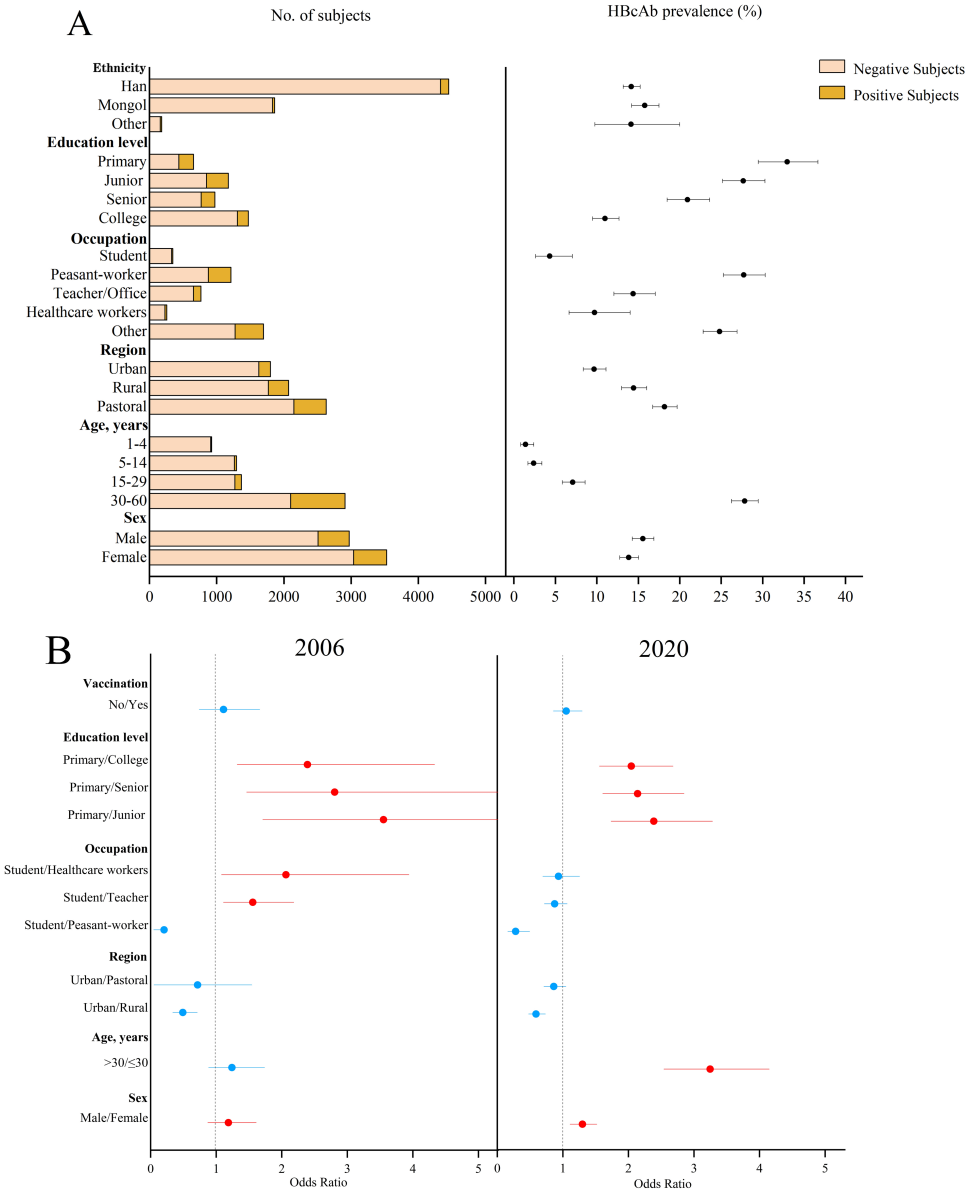
Prevalence of HBcAb in different subjects in 2020 and the possible factors related to HBcAb prevalence in 2006 and 2020 in Inner Mongolia. **(A)** The positive result and 95% CI for the prevalence of HBcAb in different subjects of Inner Mongolia in 2020. **(B)** The possible factors related to HBcAb prevalence in 2006 and 2020, including red means risk factors and blue means protective factors or vitiating factors.

The prevalence of HBcAb showed a downward trend over the period (*p* < 0.001). The prevalence of HBcAb in patients aged 1–60 years was 22.91% (95%CI: 21.87–23.94%) in 2006, which was higher than that in 2020 (20.15%, *p* = 0.01) in Figure 3, and the prevalence of HBcAb was 1.40% (95%CI: 0.82–2.40%) in patients under 5 years of age. Subjects in the urban area had the lowest prevalence of HBcAb (9.66%; 95%CI: 8.37–11.11%). The prevalence of HBcAb in students was 4.32% (95%CI: 2.62–7.06%), and the prevalence of HBcAb in subjects with a college education was 11.0% (95%CI: 9.50–12.70%). The possible factors related to the prevalence of HBcAb were male sex (OR = 1.30, 95%CI: 0.88–1.52), education level (primary vs. junior, OR = 3.55, 95%CI: 1.71–7.38; primary vs. senior, OR = 2.81, 95%CI: 1.47–5.38; primary vs. college, OR = 2.39, 95%CI: 1.32–4.33), and occupation (student vs. teacher, OR = 1.56, 95%CI: 1.11–2.18; student vs. healthcare, OR = 2.06, 95%CI: 1.08–3.94). Compared to 2006, occupation was not associated with the prevalence of HBcAb in 2020.

## 4 Discussion

HBV infection poses a formidable challenge to global health. Various preventive and control measures have been implemented to curb HBV epidemics in Inner Mongolia. Since 1992, Inner Mongolia has included the Hep B vaccine for urban newborns in its planned immunization management. In 2002, the Hep B vaccine for newborns was officially incorporated into the planned immunization plan for children (11, 12). The GAVI Project (GAVI/Children’s Vaccine Fund cooperation project) was launched in 2003. The Hep B vaccination rates in cities as a unit reached more than 80% by the end of 2005, and the TBD proportion reached more than 65% (13). In 2007, the Inner Mongolia Department of Health listed hepatitis B as a major infectious disease. The autonomous region’s finances provide subsidy funds. The Department formulated and issued the 2007–2010 Inner Mongolia Viral Hepatitis Prevention and Control Plan, settling control indicators for HBV: reducing the prevalence of HBsAg in children under 10 years old to less than 1%, reducing the prevalence of HBsAg in all subjects to less than 6%, and ensuring that the TBD vaccination rate reached more than 75% (14). The implementation plan of the Hep B vaccine project issued by the Inner Mongolia Department of Health in 2011 determined that children born between 1996 and 1997 who had not received or completed three doses of the Hep B vaccine should be vaccinated and revaccinated (15). In 2012, Tongliao City in Inner Mongolia issued the “Implementation Plan of the Project of Blocking Mother-to-Child Transmission of Hepatitis B Virus in Tongliao City,” which offered free services related to blocking mother-to-child transmission of HBV for eligible couples planning to conceive. By providing these free services, couples planning to become pregnant can access essential medical interventions and counseling, reducing the risk of mother-to-child transmission of HBV. This not only benefits individual families but also contributes to the overall public health of the region (16, 17).

### 4.1 The burden of HBV infection decreased in Inner Mongolia in 2020

In 2020, the prevalence of HBsAg in children under 5 years of age in Inner Mongolia was 0.1%, reaching the target of controlling the prevalence of HBsAg in children under 5 years of age below 1%, as mentioned in the China Viral Hepatitis Prevention and Control Program (2017–2020).

Compared to their female counterparts, males demonstrated a heightened propensity for HBV infection. This gender disparity may be attributed to several factors, such as biological differences and lifestyle (18). Understanding these sex differences is crucial for developing targeted prevention and treatment strategies. For example, health education campaigns could be tailored to address specific risk factors faced by males. Screening programs could be enhanced to ensure the early detection and treatment of HBV in high-risk groups, including males.

Compared to 2006, being over 30 years old became a risk factor for HBV infection in 2020. Furthermore, age was found to be proportional to the prevalence of HBsAg and HBcAb. Considering this, it is believed that the increase in the prevalence of HBsAg and HBcAb at a specific age is more likely to be related to the legacy of infections acquired early in life (19). This finding provides important insights into targeted preventive and control measures. For instance, intensified efforts could be directed toward augmenting awareness.

Within the older adult population and, when deemed necessary, facilitate routine screening and vaccinations. Furthermore, there is a sustained emphasis on neonatal and childhood immunization. Vaccination remains imperative to maintain low prevalence rates among these demographics and forestall the emergence of new infections.

Occupation was not found to be related to HBV infection in 2020. This change may be attributed to several reasons. Over the years, there have been significant improvements in public health education and awareness campaigns, leading to more uniform understanding and preventive measures across different occupational groups (20, 21). This showed that it was highly necessary to continue popularizing knowledge related to HBV prevention among different populations.

### 4.2 The important role of vaccination in HBV prevention and control

The Hep B vaccine has a documented positive effect on the global prevalence of HBV infection. Through extensive vaccination efforts, the prevalence of HBsAg among children under 5 years of age in the immunized child population has been reduced to less than 2%. It is estimated that by 2030, the Hep B vaccine for infants and newborns alone could prevent 2.11 million deaths (22, 23).

The effect of the HBV vaccine immunization in Inner Mongolia is remarkable. The results of two investigations have shown that Hep B vaccination is a protective factor against HBV infection. However, there were still differences in Hep B vaccine status in different regions of Inner Mongolia. The proportion of TBD and 3-dose Hep B vaccine in urban areas was higher than that in rural and pastoral areas. This may be related to incomplete vaccination due to inconvenient communication and transportation in some rural and pastoral areas, as well as loss of follow-up (24). To address this issue, efforts should be made to improve the vaccination rates in rural and pastoral areas. This could include strengthening vaccination outreach programs, ensuring better transportation of vaccines and medical personnel to these areas, and improving follow-up mechanisms to prevent the loss of contact with patients. Additionally, educational campaigns should be conducted to raise awareness about the importance of vaccination among rural and pastoral subjects.

There are still some shortcomings in this study. Compared to 2006, the proportion of Hep B vaccine use among the subjects in 2020 has increased significantly. However, the prevalence of HBsAb did not increase significantly. This is roughly the same as the research results from other provinces and cities in China, such as Tibet (25), Qinghai (26), Jiangsu (27), Jiangxi (28), Jilin (29), Shaanxi (30), and Hubei (31). In most provinces and cities of China, the prevalence of HBsAb in children aged 1–4 years is 70–85%, while the prevalence of HBsAb in children aged 5–14 years is 30–50%. This may be because the level of HBsAb decreases with time after the Hep B vaccine. This results in low antibody levels in the immunization program population. Especially after 5 years of vaccination, HBsAb decreases rapidly, leading to a relatively low level of HBsAb in the 5–17-year-old population (32). Even if HBsAb attenuates within a certain immunization period after hepatitis B vaccination, the body still has the ability to resist HBV infection (33). Therefore, the Hep B-vaccinated subjects and the immunization program subjects showed low HBsAb and HBsAg prevalence. Prior to the incorporation of hepatitis B into the immunization schedule, HBV was ubiquitously prevalent, rendering individuals highly susceptible to inadvertent exposure. Consequently, a dichotomy emerged: a subset of the population contracted HBV, manifesting as HBsAg-positive carriers, whereas others developed natural immunity, as evidenced by HBsAb positivity. This scenario precipitated a notable paradox characterized by elevated HBsAb levels amid a backdrop of high HBsAg prevalence. Given this context, a comprehensive and nuanced investigation is warranted to elucidate the current state of hepatitis B immunity in Inner Mongolia, necessitating an exploration that transcends superficial analysis.

### 4.3 Limitations of this study

First, the absolute numbers of subjects were similar between 2006 and 2020. Probably because of the large sample size, there was a statistically significant difference in the different subjects between 2006 and 2020. Second, the laboratory detection methods used in the two experiments were different. Different experimental methods may have led to a bias in the results of the two experiments. However, relevant studies have shown that the sensitivity and specificity of the two detection techniques for HBV are high (34, 35). Third, in this study, we mainly used HBsAg seroprevalence as the primary marker of HBV infection, while other biomarkers, such as HBV e antigen (HBeAg), antibodies (HBeAb), or DNA viral load, were not detected, limiting the accuracy of testing the infection status of individuals, especially those with latent infection.

## 5 Conclusion

The prevalence of HBsAg decreased significantly from 2006 to 2020 in Inner Mongolia, suggesting that the Hep B vaccine has made remarkable progress in safeguarding the population against hepatitis B infection. The HBsAg-positive proportion in Inner Mongolia differed among subjects in terms of age, region, and occupation. The prevalence of HBsAb covered by the immunization program was not high; however, the prevalence of HBsAg was low. It suggested that we should further improve Hep B vaccination efforts, supervise the TBD and 3-dose vaccination for children, and strengthen the Hep B vaccine coverage for adults. We can popularize the knowledge of HBV prevention and treatment in key areas (rural and pastoral areas) and key groups (low education level subjects and farmers). Continued efforts should be made to further reduce the burden of HBV morbidity and mortality, in line with the global goal of eliminating HBV.

## Data Availability

The original contributions presented in the study are included in the article/Supplementary material, further inquiries can be directed to the corresponding authors.
