# Supplementary material for: Changes in the prevalence of hepatitis B virus and its related factors in Inner Mongolia between 2006 and 2020
Source: Front Public Health. 2025 Apr 8;13:1533938. doi: 10.3389/fpubh.2025.1533938 (PMC12011829; doi:10.3389/fpubh.2025.1533938)
Supplement: Supplementary file 1 [file Data_Sheet_1.doc]

Table 1: Univariate logistic regression model of risk factors for HBsAg prevalence

in Inner Mongolia in 2006

| Variables |  | β | S.E | Wald χ2 | P | OR | 95%CI | |
| --- | --- | --- | --- | --- | --- | --- | --- | --- |
| Vaccination |  |  |  |  |  |  |  |  |
| No/Yes |  | 0.578 | 0.410 | 8.981 | 0.041 | 1.561 | 1.251 | 1.904 |
| Education level |  |  |  | 3.563 | 0.051 |  |  |  |
| Primary/College | | 0.551 | 0.567 | 7.116 | 0.011 | 1.735 | 0.571 | 5.272 |
| Primary/Senior | | 0.003 | 0.483 | 5.770 | 0.032 | 1.003 | 0.389 | 2.582 |
| Primary/Junior | | 0.349 | 0.465 | 2.720 | 0.113 | 1.670 | 0.908 | 3.070 |
| Occupation |  |  |  | 12.754 | 0.013 |  |  |  |
| Student/Healthcare workers | | 0.223 | 1.241 | 4.919 | 0.029 | 1.838 | 1.073 | 3.147 |
| Student/Teacher | | 0.180 | 1.109 | 13.291 | <.001 | 1.734 | 1.290 | 2.330 |
| Student/Peasant-worker | | 1.164 | 1.027 | 1.190 | 0.258 | 1.523 | 0.715 | 3.244 |
| Region |  |  |  | 23.234 | <.001 |  |  |  |
| Urban/Pastoral |  | -1.818 | 0.429 | 17.999 | <.001 | 0.162 | 0.070 | 0.376 |
| Urban/Rural | | -1.641 | 0.440 | 13.904 | <.001 | 0.194 | 0.082 | 0.459 |
| Age,years |  |  |  |  |  |  |  |  |
| >30/≤30 |  | 0.454 | 0.286 | 7.524 | 0.047 | 1.575 | 0.899 | 2.757 |
| Sex |  |  |  |  |  |  |  |  |
| Male/Female |  | 0.037 | 0.281 | 7.002 | 0.049 | 1.268 | 0.969 | 1.658 |
| Nation |  |  |  |  |  |  |  |  |
| Han/Mongol |  | -0.027 | 0.416 | 7.157 | 0.067 | 0.973 | 0.431 | 2.198 |

Table 2: Univariate logistic regression model of risk factors for HBsAg prevalence

in Inner Mongolia in 2020

| Variables |  | β | S.E | Wald χ2 | P | OR | 95%CI | |
| --- | --- | --- | --- | --- | --- | --- | --- | --- |
| Vaccination |  |  |  |  |  |  |  |  |
| No/Yes |  | 1.160 | 0.250 | 21.512 | <.001 | 3.837 | 3.188 | 4.617 |
| Education level |  |  |  | 36.299 | <.001 |  |  |  |
| Primary/College | | 1.503 | 0.274 | 30.164 | <.001 | 4.497 | 2.630 | 7.690 |
| Primary/Senior | | 0.864 | 0.257 | 11.274 | <.001 | 2.371 | 1.433 | 3.926 |
| Primary/Junior | | 0.491 | 0.223 | 4.839 | 0.028 | 1.633 | 1.055 | 2.529 |
| Occupation |  |  |  | 25.546 | <.001 |  |  |  |
| Student/Healthcare workers | | 1.094 | 1.121 | 0.952 | 0.188 | 2.985 | 0.332 | 6.870 |
| Student/Teacher | | -0.244 | 0.587 | 0.145 | 0.073 | 0.799 | 0.253 | 2.528 |
| Student/Peasant-worker | | -1.241 | 0.524 | 5.600 | 0.018 | 0.289 | 0.103 | 0.808 |
| Region |  |  |  | 20.673 | <.001 |  |  |  |
| Urban/Pastoral |  | -1.132 | 0.276 | 16.839 | <.001 | 0.322 | 0.188 | 0.553 |
| Urban/Rural | | -0.631 | 0.306 | 4.254 | 0.039 | 0.532 | 0.292 | 0.969 |
| Age,years |  |  |  |  |  |  |  |  |
| >30/≤30 |  | 0.876 | 0.236 | 13.797 | <.001 | 1.602 | 1.339 | 1.917 |
| Sex |  |  |  |  |  |  |  |  |
| Male/Female |  | 0.343 | 0.177 | 3.761 | 0.043 | 1.409 | 0.996 | 1.993 |
| Nation |  |  |  |  |  |  |  |  |
| Han/Mongol |  | -0.355 | 0.186 | 3.646 | 0.056 | 0.701 | 0.487 | 1.009 |

Table 3: Multivariable logistic regression model of risk factors for HBsAg prevalence

in Inner Mongolia in 2006

| Variables |  | β | S.E | Wald χ2 | P | OR | 95%CI | |
| --- | --- | --- | --- | --- | --- | --- | --- | --- |
| Vaccination |  |  |  |  |  |  |  |  |
| No/Yes |  | 0.439 | 0.443 | 0.981 | 0.32 | 1.551 | 0.651 | 3.696 |
| Education level |  |  |  | 1.592 | 0.66 |  |  |  |
| Primary/College | | 0.318 | 0.318 | 0.298 | 0.56 | 1.375 | 0.439 | 4.306 |
| Primary/Senior | | 0.597 | 0.597 | 0.930 | 0.34 | 1.817 | 0.540 | 6.114 |
| Primary/Junior | | 0.183 | 0.183 | 0.063 | 0.80 | 1.201 | 0.288 | 5.009 |
| Occupation |  |  |  | 6.999 | 0.14 |  |  |  |
| Student/Healthcare workers | | -0.685 | 0.688 | 0.991 | 0.32 | 0.504 | 0.131 | 1.941 |
| Student/Teacher | | -0.861 | 0.349 | 6.077 | 0.01 | 0.423 | 0.213 | 0.838 |
| Student/Peasant-worker | | -0.214 | 1.085 | 0.039 | 0.84 | 0.807 | 0.096 | 6.772 |
| Region |  |  |  | 15.150 | <.001 |  |  |  |
| Urban/Pastoral |  | -0.321 | 0.341 | 0.889 | 0.35 | 0.725 | 0.372 | 1.414 |
| Urban/Rural | | -1.707 | 0.439 | 15.132 | <.001 | 0.181 | 0.077 | 0.429 |
| Age,years |  |  |  |  |  |  |  |  |
| >30/≤30 |  | -0.378 | 0.303 | 1.557 | 0.21 | 0.685 | 0.379 | 1.241 |
| Sex |  |  |  |  |  |  |  |  |
| Male/Female |  | 0.253 | 0.295 | 0.731 | 0.03 | 1.287 | 0.721 | 2.298 |

Table 4: Multivariable logistic regression model of risk factors for HBsAg prevalence

in Inner Mongolia in 2020

| Variables |  | β | S.E | Wald χ2 | P | OR | 95%CI | |
| --- | --- | --- | --- | --- | --- | --- | --- | --- |
| Vaccination |  |  |  |  |  |  |  |  |
| No/Yes |  | 0.612 | 0.278 | 4.841 | 0.03 | 1.844 | 1.069 | 3.181 |
| Education level |  |  |  | 6.165 | 0.10 |  |  |  |
| Primary/College | | 0.254 | 0.343 | 0.548 | 0.46 | 1.289 | 0.658 | 2.526 |
| Primary/Senior | | 0.352 | 0.345 | 1.041 | 0.31 | 1.422 | 0.723 | 2.797 |
| Primary/Junior | | 0.781 | 0.367 | 4.536 | 0.33 | 2.185 | 1.064 | 4.484 |
| Occupation |  |  |  | 4.242 | 0.37 |  |  |  |
| Student/Healthcare workers | | -0.424 | 0.398 | 1.136 | 0.28 | 0.654 | 0.300 | 1.427 |
| Student/Teacher | | -0.158 | 0.220 | 0.513 | 0.47 | 0.854 | 0.555 | 1.315 |
| Student/Peasant-worker | | -0.804 | 0.560 | 2.059 | 0.15 | 0.447 | 0.149 | 1.342 |
| Region |  |  |  | 12.914 | <.001 |  |  |  |
| Urban/Pastoral |  | -0.579 | 0.233 | 6.195 | 0.01 | 0.560 | 0.355 | 0.884 |
| Urban/Rural | | -0.853 | 0.286 | 8.817 | <.001 | 0.426 | 0.243 | 0.747 |
| Age,years |  |  |  |  |  |  |  |  |
| >30/≤30 |  | 0.270 | 0.180 | 2.250 | 0.13 | 1.310 | 0.803 | 2.328 |
| Sex |  |  |  |  |  |  |  |  |
| Male/Female |  | 0.313 | 0.272 | 1.329 | 0.03 | 1.310 | 0.925 | 1.864 |

Table 5: Univariate logistic regression model of risk factors for HBsAb prevalence

in Inner Mongolia in 2006

| Variables |  | β | S.E | Wald χ2 | P | OR | 95%CI | |
| --- | --- | --- | --- | --- | --- | --- | --- | --- |
| Vaccination |  |  |  |  |  |  |  |  |
| Yes/No |  | 1.335 | 0.153 | 76.579 | <.001 | 3.798 | 2.817 | 5.121 |
| Education level |  |  |  | 12.984 | <.001 |  |  |  |
| Primary/College | | -0.582 | 0.229 | 16.467 | 0.003 | 0.955 | 0.625 | 1.460 |
| Primary/Senior | | -0.286 | 0.218 | 11.711 | 0.039 | 0.777 | 0.481 | 1.255 |
| Primary/Junior | | -0.132 | 0.219 | 10.363 | 0.044 | 0.665 | 0.370 | 1.197 |
| Occupation |  |  |  | 16.374 | <.001 |  |  |  |
| Student/Healthcare workers | | 0.507 | 0.404 | 1.575 | 0.210 | 1.660 | 0.752 | 3.664 |
| Student/Teacher | | 0.799 | 0.356 | 5.044 | 0.025 | 2.223 | 1.107 | 4.463 |
| Student/Peasant-worker | | 1.010 | 0.333 | 9.173 | 0.002 | 2.745 | 1.428 | 5.277 |
| Region |  |  |  | 24.384 | <.001 |  |  |  |
| Urban/Pastoral |  | -0.504 | 0.146 | 11.902 | <.001 | 0.604 | 0.454 | 0.804 |
| Urban/Rural | | -0.536 | 0.149 | 12.961 | <.001 | 0.585 | 0.437 | 0.783 |
| Age,years |  |  |  |  |  |  |  |  |
| >30/≤30 |  | 0.358 | 0.136 | 6.977 | 0.008 | 1.431 | 1.097 | 1.867 |
| Sex |  |  |  |  |  |  |  |  |
| Male/Female |  | 0.333 | 0.124 | 7.217 | 0.007 | 1.396 | 1.094 | 1.780 |
| Nation |  |  |  |  |  |  |  |  |
| Han/Mongol |  | -0.233 | 0.194 | 1.434 | 0.231 | 0.792 | 0.541 | 1.160 |

Table 6: Univariate logistic regression model of risk factors for HBsAb prevalence

in Inner Mongolia in 2020

| Variables |  | β | S.E | Wald χ2 | P | OR | 95%CI | |
| --- | --- | --- | --- | --- | --- | --- | --- | --- |
| Vaccination |  |  |  |  |  |  |  |  |
| Yes/No |  | 0.849 | 0.066 | 166.374 | <.001 | 2.338 | 2.055 | 2.660 |
| Education level |  |  |  | 12.984 | 0.039 |  |  |  |
| Primary/College | | -0.988 | 0.100 | 97.763 | <.001 | 0.372 | 0.306 | 0.453 |
| Primary/Senior | | -0.229 | 0.105 | 4.734 | 0.030 | 0.796 | 0.648 | 0.978 |
| Primary/Junior | | 0.002 | 0.103 | 0.000 | 0.988 | 1.002 | 0.819 | 1.225 |
| Occupation |  |  |  | 26.374 | 0.017 |  |  |  |
| Student/Healthcare workers | | 1.138 | 0.171 | 44.196 | 0.356 | 1.320 | 1.229 | 1.448 |
| Student/Teacher | | 0.535 | 0.134 | 15.943 | <.001 | 1.585 | 1.450 | 1.761 |
| Student/Peasant-worker | | 0.118 | 0.128 | 0.853 | <.001 | 1.889 | 1.692 | 2.141 |
| Region |  |  |  | 5.421 | 0.063 |  |  |  |
| Urban/Pastoral |  | -0.020 | 0.076 | 0.070 | 0.791 | 0.980 | 0.844 | 1.138 |
| Urban/Rural | | 0.389 | 0.084 | 21.208 | <.001 | 1.475 | 1.250 | 1.741 |
| Age,years |  |  |  |  |  |  |  |  |
| >30/≤30 |  | -0.220 | 0.066 | 11.019 | 0.001 | 0.246 | 0.094 | 0.489 |
| Sex |  |  |  |  |  |  |  |  |
| Male/Female |  | -0.155 | 0.063 | 6.07 | 0.014 | 0.856 | 0.757 | 0.969 |
| Nation |  |  |  |  |  |  |  |  |
| Han/Mongol |  | 0.061 | 0.070 | 0.754 | 0.385 | 1.063 | 0.926 | 1.219 |

Table 7: Multivariable logistic regression model of risk factors for HBsAb prevalence

in Inner Mongolia in 2006

| Variables |  | β | S.E | Wald χ2 | P | OR | 95%CI | |
| --- | --- | --- | --- | --- | --- | --- | --- | --- |
| Vaccination |  |  |  |  |  |  |  |  |
| Yes/No |  | 1.298 | 0.168 | 59.407 | <.001 | 3.661 | 2.632 | 5.092 |
| Education level |  |  |  | 2.984 | 0.39 |  |  |  |
| Primary/College | | -0.046 | 0.216 | 0.045 | 0.83 | 0.955 | 0.625 | 1.460 |
| Primary/Senior | | -0.252 | 0.244 | 1.065 | 0.30 | 0.777 | 0.481 | 1.255 |
| Primary/Junior | | -0.408 | 0.300 | 1.848 | 0.17 | 0.665 | 0.370 | 1.197 |
| Occupation |  |  |  | 6.374 | 0.17 |  |  |  |
| Student/Healthcare workers | | 0.293 | 0.254 | 1.330 | 0.25 | 1.340 | 0.815 | 2.203 |
| Student/Teacher | | 0.208 | 0.154 | 1.826 | 0.18 | 1.231 | 0.911 | 1.663 |
| Student/Peasant-worker | | 0.873 | 0.376 | 5.391 | 0.02 | 2.394 | 1.146 | 5.003 |
| Region |  |  |  | 25.421 | 0.46 |  |  |  |
| Urban/Pastoral |  | -0.072 | 0.180 | 0.161 | 0.69 | 0.750 | 0.056 | 1.528 |
| Urban/Rural | | -0.689 | 0.159 | 18.857 | 0.23 | 0.502 | 0.368 | 0.685 |
| Age,years |  |  |  |  |  |  |  |  |
| >30/≤30 |  | -0.096 | 0.154 | 0.384 | 0.54 | 0.909 | 0.813 | 1.489 |
| Sex |  |  |  |  |  |  |  |  |
| Male/Female |  | 0.199 | 0.136 | 2.151 | 0.14 | 1.220 | 0.935 | 1.592 |

Table 8: Multivariable logistic regression model of risk factors for HBsAb prevalence

in Inner Mongolia in 2020

| Variables |  | β | S.E | Wald χ2 | P | OR | 95%CI | |
| --- | --- | --- | --- | --- | --- | --- | --- | --- |
| Vaccination |  |  |  |  |  |  |  |  |
| Yes/No |  | 0.724 | 0.077 | 87.885 | <.001 | 2.064 | 1.774 | 2.401 |
| Education level |  |  |  | 11.598 | 0.01 |  |  |  |
| Primary/College | | -0.261 | 0.100 | 6.857 | 0.01 | 0.770 | 0.633 | 0.936 |
| Primary/Senior | | -0.355 | 0.108 | 10.733 | <.001 | 0.702 | 0.567 | 0.867 |
| Primary/Junior | | -0.298 | 0.130 | 5.250 | 0.02 | 0.742 | 0.575 | 0.958 |
| Occupation |  |  |  | 20.230 | <.001 |  |  |  |
| Student/Healthcare workers | | -0.056 | 0.107 | 0.279 | 0.60 | 0.945 | 0.766 | 1.165 |
| Student/Teacher | | -0.225 | 0.088 | 6.471 | 0.04 | 0.746 | 0.566 | 0.984 |
| Student/Peasant-worker | | 0.292 | 0.141 | 4.302 | 0.01 | 1.252 | 1.053 | 1.489 |
| Region |  |  |  | 23.915 | <.001 |  |  |  |
| Urban/Pastoral |  | -0.404 | 0.083 | 23.814 | <.001 | 0.668 | 0.567 | 0.785 |
| Urban/Rural | | -0.147 | 0.081 | 3.295 | 0.07 | 0.864 | 0.737 | 1.012 |
| Age,years |  |  |  |  |  |  |  |  |
| >30/≤30 |  | -0.035 | 0.080 | 0.197 | 0.66 | 0.965 | 0.825 | 1.129 |
| Sex |  |  |  |  |  |  |  |  |
| Male/Female |  | -0.086 | 0.066 | 1.712 | 0.19 | 0.918 | 0.807 | 1.044 |

Table 9: Univariate logistic regression model of risk factors for HBcAb prevalence

in Inner Mongolia in 2006

| Variables |  | β | S.E | Wald χ2 | P | OR | 95%CI | |
| --- | --- | --- | --- | --- | --- | --- | --- | --- |
| Vaccination |  |  |  |  |  |  |  |  |
| No/Yes |  | 0.134 | 0.185 | 5.524 | 0.046 | 1.874 | 1.608 | 2.257 |
| Education level |  |  |  | 12.384 | <.001 |  |  |  |
| Primary/College | | 1.182 | 0.276 | 18.382 | <.001 | 3.262 | 1.900 | 5.599 |
| Primary/Senior | | 0.505 | 0.231 | 4.799 | 0.028 | 1.657 | 1.055 | 2.605 |
| Primary/Junior | | 0.231 | 0.225 | 1.057 | 0.304 | 1.260 | 0.811 | 1.957 |
| Occupation |  |  |  | 13.421 | 0.032 |  |  |  |
| Student/Healthcare workers | | -1.125 | 0.809 | 1.933 | 0.164 | 0.325 | 0.067 | 1.585 |
| Student/Teacher | | -1.290 | 0.755 | 2.919 | 0.008 | 0.275 | 0.063 | 1.209 |
| Student/Peasant-worker | | -1.999 | 0.732 | 7.458 | 0.006 | 0.136 | 0.032 | 0.569 |
| Region |  |  |  | 18.336 | <.001 |  |  |  |
| Urban/Pastoral |  | -0.788 | 0.183 | 18.584 | <.001 | 0.455 | 0.318 | 0.651 |
| Urban/Rural | | -1.059 | 0.181 | 34.327 | <.001 | 0.347 | 0.243 | 0.494 |
| Age,years |  |  |  |  |  |  |  |  |
| >30/≤30 |  | 0.189 | 0.160 | 7.410 | 0.023 | 1.209 | 0.884 | 1.653 |
| Sex |  |  |  |  |  |  |  |  |
| Male/Female |  | 0.231 | 0.146 | 12.509 | 0.013 | 1.260 | 0.947 | 1.678 |
| Nation |  |  |  |  |  |  |  |  |
| Han/Mongol |  | -0.156 | 0.225 | 0.482 | 0.487 | 0.855 | 0.551 | 1.329 |

Table 10: Univariate logistic regression model of risk factors for HBcAb prevalence

in Inner Mongolia in 2020

| Variables |  | β | S.E | Wald χ2 | P | OR | 95%CI | |
| --- | --- | --- | --- | --- | --- | --- | --- | --- |
| Vaccination |  |  |  |  |  |  |  |  |
| No/Yes |  | 0.687 | 0.087 | 62.995 | <.001 | 1.503 | 1.425 | 1.596 |
| Education level |  |  |  | 87.651 | <.001 |  |  |  |
| Primary/College | | 1.382 | 0.118 | 137.915 | <.001 | 3.982 | 3.162 | 5.014 |
| Primary/Senior | | 0.619 | 0.114 | 29.235 | <.001 | 1.857 | 1.484 | 2.324 |
| Primary/Junior | | 0.252 | 0.106 | 5.698 | 0.017 | 1.287 | 1.046 | 1.583 |
| Occupation |  |  |  | 113.421 | <.001 |  |  |  |
| Student/Healthcare workers | | -0.869 | 0.338 | 6.628 | 0.010 | 0.419 | 0.216 | 0.813 |
| Student/Teacher | | -1.313 | 0.283 | 21.468 | <.001 | 0.269 | 0.154 | 0.469 |
| Student/Peasant-worker | | -2.139 | 0.272 | 61.989 | <.001 | 0.118 | 0.069 | 0.201 |
| Region |  |  |  | 77.331 | <.001 |  |  |  |
| Urban/Pastoral |  | -0.735 | 0.102 | 51.433 | <.001 | 0.479 | 0.392 | 0.586 |
| Urban/Rural | | -0.625 | 0.110 | 32.229 | <.001 | 0.535 | 0.431 | 0.664 |
| Age,years |  |  |  |  |  |  |  |  |
| >30/≤30 |  | 1.619 | 0.113 | 204.761 | <.001 | 1.198 | 1.159 | 2.247 |
| Sex |  |  |  |  |  |  |  |  |
| Male/Female |  | 0.303 | 0.075 | 16.305 | <.001 | 1.354 | 1.169 | 1.569 |
| Nation |  |  |  |  |  |  |  |  |
| Han/Mongol |  | -0.208 | 0.082 | 0.401 | 0.110 | 0.813 | 0.692 | 0.954 |

Table 11: Multivariable logistic regression model of risk factors for HBcAb prevalence

in Inner Mongolia in 2006

| Variables |  | β | S.E | Wald χ2 | P | OR | 95%CI | |
| --- | --- | --- | --- | --- | --- | --- | --- | --- |
| Vaccination |  |  |  |  |  |  |  |  |
| No/Yes |  | 0.106 | 0.206 | 0.262 | 0.61 | 1.111 | 0.742 | 1.664 |
| Education level |  |  |  | 12.066 | 0.01 |  |  |  |
| Primary/College | | 0.872 | 0.303 | 8.281 | <.001 | 2.392 | 1.321 | 4.331 |
| Primary/Senior | | 1.032 | 0.332 | 9.684 | <.001 | 2.808 | 1.465 | 5.379 |
| Primary/Junior | | 1.268 | 0.373 | 11.559 | <.001 | 3.553 | 1.711 | 7.380 |
| Occupation |  |  |  | 15.403 | <.001 |  |  |  |
| Student/Healthcare workers | | 0.724 | 0.330 | 4.822 | 0.03 | 2.064 | 1.081 | 3.940 |
| Student/Teacher | | 0.443 | 0.173 | 6.591 | 0.01 | 1.557 | 1.110 | 2.184 |
| Student/Peasant-worker | | -1.581 | 0.756 | 4.370 | 0.04 | 0.206 | 0.047 | 0.331 |
| Region |  |  |  | 18.700 | <.001 |  |  |  |
| Urban/Pastoral |  | -0.049 | 0.196 | 0.063 | 0.80 | 0.716 | 0.056 | 1.542 |
| Urban/Rural | | -0.712 | 0.190 | 14.093 | <.001 | 0.491 | 0.338 | 0.711 |
| Age,years |  |  |  |  |  |  |  |  |
| >30/≤30 |  | 0.215 | 0.172 | 1.555 | 0.21 | 1.240 | 0.884 | 1.737 |
| Sex |  |  |  |  |  |  |  |  |
| Male/Female |  | 0.170 | 0.156 | 1.186 | 0.03 | 1.298 | 0.882 | 1.518 |

Table 12: Multivariable logistic regression model of risk factors for HBcAb prevalence

in Inner Mongolia in 2020

| Variables |  | β | S.E | Wald χ2 | P | OR | 95%CI | |
| --- | --- | --- | --- | --- | --- | --- | --- | --- |
| Vaccination |  |  |  |  |  |  |  |  |
| No/Yes |  | 0.051 | 0.105 | 0.234 | 0.63 | 1.052 | 0.856 | 1.293 |
| Education level |  |  |  | 34.744 | <.001 |  |  |  |
| Primary/College | | 0.715 | 0.138 | 26.700 | <.001 | 2.044 | 1.558 | 2.680 |
| Primary/Senior | | 0.761 | 0.146 | 27.206 | <.001 | 2.140 | 1.608 | 2.848 |
| Primary/Junior | | 0.870 | 0.162 | 28.686 | <.001 | 2.387 | 1.736 | 3.282 |
| Occupation |  |  |  | 20.217 | 0.05 |  |  |  |
| Student/Healthcare workers | | -0.069 | 0.151 | 0.210 | 0.65 | 0.933 | 0.694 | 1.255 |
| Student/Teacher | | -0.134 | 0.100 | 1.801 | 0.18 | 0.875 | 0.719 | 1.064 |
| Student/Peasant-worker | | -1.272 | 0.288 | 19.466 | 0.06 | 0.280 | 0.159 | 0.493 |
| Region |  |  |  | 23.100 | <.001 |  |  |  |
| Urban/Pastoral |  | -0.148 | 0.099 | 2.259 | 0.13 | 0.862 | 0.711 | 1.046 |
| Urban/Rural | | -0.525 | 0.109 | 23.040 | <.001 | 0.591 | 0.477 | 0.733 |
| Age,years |  |  |  |  |  |  |  |  |
| >30/≤30 |  | 1.177 | 0.125 | 88.915 | <.001 | 3.246 | 2.541 | 4.146 |
| Sex |  |  |  |  |  |  |  |  |
| Male/Female |  | 0.051 | 0.105 | 0.234 | 0.03 | 1.052 | 0.856 | 1.593 |
